# 

**Published:** 1912-06-29

**Authors:** 


### OUR BUREAU OF INFORMATION.

### Rules for Correspondents.

1. Every letter, on whatever subject, must be accompanied by the coupon to be cut from the back cover (inside page) of The Hospital, current issue, and must contain the name and address of the correspondent with pseudonym for publication if desired.

2. Replies by post cannot be given, save under exceptional circumstances, at the Editor's discretion.

3. Letters from Approved Homes in reply to special needs published in the Bureau should state terms and full particulars, and be sent prepaid under cover to the Editor of the Bureau with coupon and name enclosed for identification.

4. Proprietors of homes which have not yet been entered on the List of Approved Homes, but have spare accommodation likely to suit special needs, are invited to write for an application form for registration. The fee for registration is 5s.

5. Special needs of every description relating to those who are sick or in difficulties are made known in these columns free of charge.

6. All communications to be addressed to The Editor of The Hospital, 28 Southampton Street, Strand, London, W.C., and marked "Bureau of Information."

### Hospital Management.

### Women on Hospital Boards.

Eunice.—There is a great difference of opinion among hospital authorities in regard to the presence of women on hospital boards. It is far more a question of expediency than of principle. If and when women properly qualified for the business to be transacted can be selected, their services are likely to be of great value, but it is highly expert work, which few women have studied. The appointment of a ladies' committee is usually a more satisfactory way of securing the help which women are qualified to give in hospital matters. The Royal Free is the only London general hospital which has made the experiment of admitting women to its board. In the provinces and in Scotland and Wales the practice is fairly common, and in many children's and special hospitals.

### New Legislation.

### Powers to Detain Defectives.

Freedom.—Under the Government Mental Deficiency Bill safeguards are introduced against the detention through malice or interested motives of persons of sound mind. The dread of interference with the liberty of the subject which you express is responsible for the *laissez-faire* policy of which we are beginning to behold the deplorable results in the unrestricted propagation of the unfit. Is it not far more cruel to allow persons of feeble intellect to drift in and out of the workhouse in a degraded condition than to confine them in an environment suited to their intelligence in which they can pass lives freed from gross temptation?

### Homes Wanted.

### After-Care Home in Ireland.

Mrs. L. Mullaghmast (111a).—Home wanted (near Dublin preferred) for lady recovering from mental trouble and at present in a mental hospital. The terms offered are too low. Write, however, to Nurse Roberts-Murray, Rosebank, Herbert Road, Bray, Co. Wicklow, who will meet you as regards terms to the best of her ability. Prepaid replies sent under cover of the Bureau will be forwarded.

### For Infant.

Mrs. R., Chelsea (112a).—You do not state what payment could be made for the baby, if any. If you will write us fully in confidence we may be able to suggest an opening for the young mother when she is well enough to work, and could certainly find a home for the infant, but some payment must be guaranteed, even if it is adopted.

### Massage and Medical Gymnastics.

### The Swedish versus the German Method.

L. G. T., York.—The Swedish methods have been generally adopted in this country and are more likely to be of service in procuring appointments. You cannot learn from books without a regular course of training. The German system is far more varied, and as it permits the use of music and a great variety of appliances it is more attractive from a child's point of view. Each system requires two years' training, but to learn them both as can be done at some colleges takes three years.

### Sanitation.

### Mosquitoes as Disease Carriers.

Colonial.—It is impossible to say, unless you send us a specimen, whether the gnats which infest your compound are disease carriers or not. The systematic study of the Culicidæ only dates from 1899, but already 100 genera and some 700 distinct species have been identified. There are at least 100 species among the Anophelinæ, which are distinguished by narrow bodies, spotted wings, and long palpi. These are known to disseminate malaria; the thirst for blood is stimulated by heat, but is for the most part confined to the females. The mosquito known as Stegomyia Calopus, the carrier of yellow fever, belongs to another genus, the Culicinæ. Filariasis is disseminated by both these genera. The preliminary stages of all mosquitoes are passed in stagnant water, and their range is said, though with doubtful exactitude, to be limited to three hundred yards, so you should be able to procure almost complete immunity from their attacks.

### The Income of the Hospitals.

### The League of Mercy.

Half-Pay.—The League of Mercy was founded in 1899 for the purpose of raising money for the London hospitals in contributions of one shilling a year and upwards. Its work has now extended to the home counties. The grants distributed by the League in 1911 reached a total of £19,200, and if that year be included the amounts collected since its beginning are considerably over £200,000. Honorary assistance is welcomed, and if willing to help you should apply to the Secretary, League of Mercy Offices, 28 and 29 Southampton Street, Strand, London, W.C.

### The Revenue of British Charities.

Nurse B., Hamburg.—You may tell your critic that in 1910 the income from all sources of our charitable institutions was £12,455,000. About 40 per cent. of this amount was received by institutions for the care and cure of the sick, 20 per cent. for home and foreign missions, and the remainder, 32 per cent., for orphanages and other homes and charities. These facts speak for themselves.

### Letter-Box.

F. M. R., Ilfracombe.—We have forwarded your letter to the home, and doubtless you will hear in due course whether there is a vacancy at the time you name.

Mrs. R.—Many thanks for the forms received.

H. O. B. (102x).—We are making arrangements to have the lady visited.

### Appointments.

The Mental Hospitals Committee of the London County Council have recently appointed Colonel R. J. Cooper to be a member of the Claybury and Hanwell Mental Hospitals Sub-Committees, and Mr. E. G. Easton to be a member of the Cane Hill Mental Hospital Sub-Committee.

The following appointments at the Manchester Royal Infirmary have been confirmed :—To be assistant medical officers : Dr. E. B. Leech, M.R.C.P.; Dr. C. H. Molland, M.R.C.P.; and Dr. F. E. Tylecote, M.R.C.P. To be assistant director of clinical laboratory : Dr. R. Briercliffe, M.B., Ch.B. (Vict.); while Dr. John Gow, M.B., Ch.B. (Vict.), has been appointed accident-room house surgeon.

### Buildings Contemplated.

Ballylesson.—The Lisburn Guardians have decided to apply to the Local Government Board for sanction to a loan of £1,900 for the purchase of a site, together with the erection of a dispensary and medical officer's residence.

Barking.—A tender of £1,820 has been accepted by the Ubran District Council for an administrative block at Upney Hospital.

Blaby.—The Guardians have accepted a tender of £2,098 for structural alterations at the workhouse.

Bury (Lancs).—At the Aitken Sanatorium, Holcombe, it is proposed to install electric light and construct a bore-hole pump. Particulars may be had from Mr. S. J. Watson, M.I.E.E., Electricity Works, Bury, on deposit of £1 1s.

Cardiff.—The Local Government Board have held an inquiry into an application of the Corporation for sanction to a borrowing of £2,200 in regard to the City Mental Hospital. Dr. Goodall, the medical superintendent at Whitchurch, has prepared the scheme.—Mr. E. Seward, F.R.I.B.A., Cardiff, is architect for sundry alterations and additions to the Ely Workhouse. Tenders are invited, and particulars may be had from the architect. A bond of £50 and two sureties are required for the due performance of the contact.

Clatterbridge (Cheshire).—Nine tenders have been received for erection of master's house, children's block, and remodelling the laundry at Clatterbridge Workhouse for the Wirral Guardians. The lowest is £3,193, the highest £3,607. Messrs. Davies and Son, of Chester, are the architects.

Dewsbury.—Messrs. W. Hanstock and Son, of Batley, are architects for the erection of a new phthisical ward at the workhouse infirmary. Tenders are invited, and particulars may be had from the architects.

Etwall (Derby).—Mr. R. W. Sanders, of Repton, is architect for extensions at the Etwall Isolation Hospital for the Repton Committee. The work includes the provision of a fever ward verandah and new floors to the diphtheria block and offices. Tenders are invited.

Hendon.—The Hendon and the Finchley Urban District Councils are making a joint application for sanction to purchase six acres of land at Renter's Lane, Hendon, for an isolation hospital.

Hertford.—Plans have been approved for remodelling the Hertford County Hospital at an estimated cost of £10,000.

Kettering.—The Kettering Joint Hospital Board has decided to apply to the Local Government Board for sanction to a loan to cover additions to the hospital.

Llysfain.—Alderman F. J. Gamlin, of Rhyl, has offered a free site of 22 acres at Llysfain, near Rhyl, for a phthisis sanatorium.

Park Prewett.—The joint asylum committee appointed by the Hampshire County Council have accepted a tender of £32,050 and £4,348 respectively for foundation works and for a railway to connect the site with the L. and S.W. system.

Pontefract.—Tenders amounting to £2,375 have been accepted for a new pavilion and other additions, etc., at the Pontefract Joint Isolation Hospital. Messrs. Garside and Pennington are the architects.

Salisbury.—The Wilts County Council are making application for sanction to a loan of £2,750 for the erection and equipment of an isolation hospital.

Southend-on-Sea.—It is proposed to erect a new administrative block at the Southend-on-Sea Sanatorium, estimated to cost about £4,000. A design has been prepared by Mr. E. J. Elford, M.Inst.C.E., the Borough Surveyor, and tender forms may be obtained on application to him.

Sunderland.—Messrs. W. and T. R. Milburn are architects for the new Children's Hospital which has just been formally opened. The cost of the works was £21,000, and accommodation is provided for fifty-four patients.

Swinton.—The Manchester Guardians propose to erect new reception wards at an estimated cost of £9,100.

Warrington.—Messrs. W. and S. Owen have prepared plans for additional accommodation for mental and epileptic patients at the Guardians' premises. The cost of the buildings is estimated at £12,666.

Willesden.—The Willesden Guardians invite tenders for the erection of children's homes in Acton Lane. Particulars may be had from the architect, Mr. J. Cash, F.R.I.B.A., 22 Surrey Street, W.C., upon deposit of a £20 note.

Winsley.—Alterations and additions are projected for the Winsley Sanatorium, near Bath. Mr. W. S. Skinner, of Bristol, is architect, and will supply information for tenders. A deposit of £2 2s. is required.

Correction.—Page 312 of our issue of June 22 : Proposed Cottage Hospital, Haines, should read Staines.

### THE SEASON AT BADEN-BADEN.

The season at Baden-Baden has now begun, and visitors are arriving in search of health and pleasure at this spa, which has been called "The Queen of the Black Forest." The well-known Lichtenthaler-Allée presents an animated picture in the afternoon. The spring holidays were favoured with magnificent weather, and the programme for this month, which many consider the best to spend in Germany, is a varied and interesting one. Concerts, *fêtes* at the Old Castle, Nuits Vénétiennes, and motor-car excursions in the Black Forest cater for the different tastes of holiday-makers. The woods just now are most luxuriant. The Karlsruhe Theatrical Company will give performances at the theatre. The Zeppelin airship "Schwaben" makes daily trips from Baden-Baden, and is much patronised. From time to time visitors taking strolls in the Gardens are surprised by a humming sound in the air, and are unable to offer an explanation until they see almost over their heads the cigar-shaped "Schwaben" passing in one direction or the other.

### Further Answers to Insurance Questions.*

A. B.—Is the 7s. 6d. sickness benefit paid in full to domestic servants while they are being boarded and lodged by their mistress, or does the original clause in the Bill still stand which forbade payment under the circumstances?—The sickness benefit *is* payable even though the servant is being boarded and lodged by her mistress. An employer is not relieved from any legal liability to pay wages during sickness to any person employed by him (or her) in accordance with any established custom (Section 47 (12)). The servant may voluntarily surrender the benefit to her mistress, but any agreement that may be made to this effect is void (Section 111).

Puzzled.—Is a boy who is engaged to clean boots, etc., and receives 8s. a week and partial board, such as breakfast and tea, to be insured, and, if so, what would the premiums be, and is this irrespective of what his age may be?—As the employment is regular the boy will have to be insured, provided he is over sixteen years of age. The contribution payable by the employer will be 7d. a week, and he (or she) will be entitled to deduct 4d. a week from the wage paid in all cases, if any monetary payment is made, if the age of the employee is not more than twenty-one. Above age of twenty-one the rate of remuneration must be taken into account, and a value will be set on the partial board provided, but the variations probably do not apply on account of the age restriction to the case in question.

Passive Resister.—Why do you not mention that every servant when out of a place has to pay the employer's 3d. as well as her own? Is it not true that when no money is coming in the servant has to pay twice what she does when earning a salary?—When out of employment payment of contribution is optional. If the contributions are not paid in full—*i.e.*, the employer's 3d. as well as the employee's 3d.—the servant may suffer. No reduction will be made in the benefits until the contributions are more than three weeks on the average for each year insured in arrear. To obviate the reduction when the arrears exceed this sum the servant is allowed to pay up the arrears after she returns to work so far as those arrears have accrued during the current and the preceding calendar year. If she is a member of an approved society the society may, if it chooses, excuse the arrears so far as the portion that would have been paid by the employer. We deal with this fully in our article on "Arrears of Contribution."

* The first batch of Insurance Questions and Answers appears on page 324.

### Gifts and Bequests.

Mr. George Baxter, of Shepherd's Bush, W., has left £1,000 each to the Westminster Hospital, the Middlesex Hospital, the West London Hospital, Hammersmith, and the National Hospital for the Paralysed and Epileptic, Queen Square, Bloomsbury, W.C., in each case for the endowment of a bed.

Mr. Thomas Stephen Whitaker, of Everthorpe Hall, East Yorkshire, has left £64,000 to charities, including £5,000 each to the Hull Royal Infirmary, Hull and Sculcoates Dispensary, Victoria Hospital for Children, Chelsea, the Hospital for Consumption and Diseases of the Chest, Fulham Road, London, the London Fever Hospital, Islington, and £1,000 to the Norfolk and Lynn Hospital, King's Lynn.

The Rev. James Marshall, of South Hampstead, N.W., has bequeathed £500 to the Westminster Hospital.

The Chelsea Hospital for Women has received donations of £500 from Mr. Henry E. Wright and 50 guineas from the Worshipful Company of Drapers.

### Church Rivalry on Hospital Sunday.

The following are among the amounts received at the Mansion House to date: William Herring, Esq., £1,000; St. Michael's, Chester Square, £608; Heath Harrison, Esq., £250; St. Paul's, Onslow Square, £249; Essex Unitarian Church, Kensington, £245; St. Paul's Cathedral, £227; Messrs. Watts, Watts and Co., £210; St. Columba's (Church of Scotland), Pont Street, £320; Holy Trinity, Brompton, £210; Alexander Miller, Esq., £200; "F. G. D.," £200; Westminster Chapel, £193; St. Jude's, South Kensington, £176; The Theistic Church, Swallow Street, St. James's, per Rev. C. Voysey, £175; St. James's, Piccadilly, £163; St. Margaret's, Westminster, £140; St. Peter's, Vere Street, £133; St. Simon's, Upper Chelsea, £133; Emmanuel, Wimbledon, £121; St. George's, Bickley, £120; St. Nicholas, Chislehurst, £120; City Temple, £119; Bromley Parish Church and Chapel of Ease, £118; Hampstead Unitarian Church, £117; Temple Church, £115; St. John, Penge, £108; Union Chapel, Islington, £103; St. John's, Paddington, £103; St. Peter's, Brockley, £101; Lieut.-Colonel E. More-Nisbett, £100; Alderman Sir W. Vaughan Morgan, Bart., £100; W. D. Graham-Menzies, Esq., £100; Mrs. Foster, £100; Field-Marshal Sir Chas. Brownlow and Lady Brownlow, £100; Brixton Independent Church, £100; St. Mark's, Reigate, £100; Sir John Ramsden, £100; St. Mary, Boltons, £99; St. Paul's, Westbourne Grove, £93; St. Mark's, Hamilton Terrace, £91; Roehampton Parish Church, £87; Christ Church, Chislehurst, £86; Chipping Barnet Parish Church, £82; St. Anne's, Wandsworth, £82; All Saints', Ennismore Gardens, £81; St. Matthew's, Bayswater, £78; St. James's, Paddington, £78; Beckenham Parish Church, £75; The Oratory, South Kensington, £74; Greek Church, Bayswater, £73; St. Anne's, Soho, £69; St. Philip, Kensington, £68; St. Andrew's Presbyterian Church, Frognal, £67; Holy Trinity, Eltham, £67; St. Michael, Blackheath, £63; Immanuel, Streatham, £62; St. James, Kidbrook, £59; Christ Church, Beckenham, £58; Christ Church, Lee, £58; St. Magnus-the-Martyr, London Bridge, £56; Christ Church, Victoria Street, £56; St. Paul's, Wimbledon, £55; Lemsford Parish Church, £54; Messrs. J. H. Vavasseur and Co., £52; Weybridge Churches, £51; Mrs. C. E. Layton, £50; Mrs. Kenneth M. Clark, £50; "A Friend," £50; G. P. Gooch, Esq., £50; "A. B. C.," £50; "In Memoriam R. G. S.," £50; St. James, Muswell Hill, £49; St. Augustine's, Highbury, £47; Dutch Church, Austin Friars, £47; Crouch Hill Presbyterian Church, £45; St. Stephen's with St. Luke's, Ealing, £45; St. Gregory, Earlsfield, £45; Allen Street Congregational, Kensington, £44; St. Augustine's, Kilburn, £44; St. James, Garlickhithe, £43; St. George, Perry Hill, £43; Christ Church, Newgate Street, £43; Chapel Royal, Savoy, £43; St. Stephen's, Clapham, £42; St. Mary, Graham Street, £42; St. John, Putney, £42; Carmelite Church, Kensington, £40; St. Stephen's, Westminster, £40; Gray's Inn Chapel, £37; Ilford Parish Church and Chapel of Ease, £37; St. John's, Blackheath, £37; St. Mary, Shortlands, £38; St. Mary Magdalene, Wandsworth Common, £36; Chapel Royal, St. James's, £36.

